# Genotyping of *Streptococcus agalactiae *(group B streptococci) isolated from vaginal and rectal swabs of women at 35-37 weeks of pregnancy

**DOI:** 10.1186/1471-2334-9-153

**Published:** 2009-09-11

**Authors:** Nabil Abdullah El Aila, Inge Tency, Geert Claeys, Bart Saerens, Ellen De Backer, Marleen Temmerman, Rita Verhelst, Mario Vaneechoutte

**Affiliations:** 1 Laboratory Bacteriology Research, Department of Clinical Chemistry, Microbiology & Immunology, University of Ghent, Ghent, Belgium; 2 Department of Obstetrics & Gynaecology, Ghent University Hospital, University of Ghent, Ghent, Belgium

## Abstract

**Background:**

Group B streptococci (GBS), or *Streptococcus agalactiae*, are the leading bacterial cause of meningitis and bacterial sepsis in newborns. Here we compared different culture media for GBS detection and we compared the occurrence of different genotypes and serotypes of GBS isolates from the vagina and rectum.

**Methods:**

*Streptococcus agalactiae *was cultured separately from both rectum and vagina, for a total of 150 pregnant women, i) directly onto Columbia CNA agar, or indirectly onto ii) Granada agar resp. iii) Columbia CNA agar, after overnight incubation in Lim broth.

**Results:**

Thirty six women (24%) were colonized by GBS. Of these, 19 harbored GBS in both rectum and vagina, 9 only in the vagina and 8 exclusively in the rectum. The combination of Lim broth and subculture on Granada agar was the only culture method that detected all GBS positive women. Using RAPD-analysis, a total of 66 genotypes could be established among the 118 isolates from 32 women for which fingerprinting was carried out. Up to 4 different genotypes in total (rectal + vaginal) were found for 4 women, one woman carried 3 different genotypes vaginally and 14 women carried two 2 different genotypes vaginally. Only two subjects were found to carry strains with the same genotype, although the serotype of both of these strains was different.

Eighteen of the 19 subjects with GBS at both sites had at least one vaginal and one rectal isolate with the same genotype.

We report the presence of two to four different genotypes in 22 (61%) of the 36 GBS positive women and the presence of identical genotypes in both sites for all women but one.

**Conclusion:**

The combination of Lim broth and subculture on Granada medium provide high sensitivity for GBS detection from vaginal and rectal swabs from pregnant women. We established a higher genotypic diversity per individual than other studies, with up to four different genotypes among a maximum of 6 isolates per individual picked. Still, 18 of the 19 women with GBS from both rectum and vagina had at least one isolate from each sampling site with the same genotype.

## Background

*Streptococcus agalactiae*, group B *Streptococcus *(GBS), is a leading cause of neonatal morbidity and mortality in the US, Western Europe and Australia. Maternal carriage has been recognized as the most important risk factor for GBS neonatal infection [1,2] and indeed vertical transmission before or during delivery has been shown [3,4]. Mother-to-child transmission may lead to neonatal infection in 1 to 2 infants per 1,000 live births [5] with mortality rates ranging from 10 to 20% [6]. Among pregnant women, the prevalence of colonization with GBS ranges from 3.2 to 36% [7-9]. Screening consists of obtaining vaginal and rectal specimens for culture at 35 to 37 weeks of gestation. Recently, several molecular techniques have been applied to study the genetic diversity of GBS, such as restriction fragment length polymorphism analysis (RFLP) [10], ribotyping [10,11], pulsed-field gel electrophoresis (PFGE) [3,12-17] multilocus enzyme electrophoresis (MLEE) [18] randomly amplification of polymorphic DNA-analysis (RAPD) [9,19,20], amplified *cps *restriction polymorphism analysis [21] and multilocus sequence typing (MLST) [13,22-25].

To our knowledge, only one study [12], addressed the genotypic and serological diversity of GBS within individual women. Therefore, the aim of this study was to compare the genotypes of the GBS isolates from separate vaginal and rectal swabs using a simple genotypic approach (RAPD analysis with primer OPM1, followed by capillary electrophoresis) and also to study the correlation between serotype and genotype of the GBS isolates.

## Methods

### Study design

The study was approved by the research ethics committee (IRB protocol nr 2007/096) of Ghent University Hospital, Flanders, North region of Belgium, and all the women gave written informed consent. Between April and December 2007, 150 paired vaginal and rectal swabs were collected from pregnant women at 35 - 37 weeks of gestation.

### Collection and culture of specimens

All specimens were collected using nylon flocked swabs that were submerged into 1 ml of liquid Amies transport medium (eSwab, Copan Diagnostics, Brescia, It.). For rectal specimens, a swab was carefully inserted approximately 1.5 - 2 cm beyond the anal sphincter and then gently rotated to touch anal crypts.

Vaginal samples were collected by inserting a swab into the vagina. The swab was rolled round through 360 degrees against the vaginal wall at the midportion of the vault. At Ghent University Hospital, the routine GBS-screening of pregnant women is always performed during the prenatal consultation at 35-37 weeks' gestation. All study samples were collected by midwives and transported to the Laboratory of Bacteriology Research within 4 hours.

A total of 70 μl from the Amies liquid transport medium of each of the vaginal and rectal swabs was seeded on Columbia CNA agar with 5% sheep blood (Columbia CNA agar, Becton Dickinson, Erembodegem, Belgium) and 200 μl was inoculated into 5 ml of Lim Broth (Todd-Hewitt broth, 1% yeast extract, 15 μg nalidixic acid/ml and 10 μg colistin/ml) (Lim Broth, Becton Dickinson) [26]. Both media are selective for Gram positive bacteria.

The Columbia CNA agar plates were incubated at 37°C in 5% CO_2 _for 24-48 h.

The Lim Broth was incubated aerobically at 37°C for 18-24 hours and then subcultured onto Granada agar (Becton Dickinson) [27] and onto Columbia CNA agar. Granada agar was incubated at 37°C in an anaerobic chamber (BugBox, LedTechno, Heusden-Zolder, B.) for 24-48 h and Columbia CNA agar was incubated at 37°C in 5% CO_2 _for 24-48 h.

Granada agar was examined for yellow-orange pigment colonies that confirm the presence of GBS, whereas β-haemolytic and non-haemolytic colonies were picked from Columbia CNA agar for further identification as *S. agalactiae *by the CAMP test.

### Identification of the isolates as *Streptococcus agalactiae*

The isolates were identified as *S. agalactiae *by the following criteria: growth and orange pigment formation on Granada agar, positive for the CAMP test on blood agar and molecular identification by tDNA-PCR [28].

### Antibiotic susceptibility testing

Fourty isolates of group B streptococci of 8 pregnant women were tested by disk diffusion for susceptibility to clindamycin and erythromycin Colonies taken from Trypticase Soy Agar (TSA) + 5% sheep blood (Becton Dickinson) were suspended in 5 ml of saline and the inoculum was adjusted to the turbidity of a 0.5 McFarland standard. This suspension was streaked onto TSA + 5% sheep blood to obtain confluent growth, disks were added and the plates were incubated overnight at 37°C with 5% CO_2_. Strains were considered resistant to clindamycin and erythromycin when the inhibition zones were less than 15 mm.

### DNA-extraction from isolates

DNA was extracted from cultured isolates by alkaline lysis as follows: One bacterial colony was suspended in 20 μl of lysis buffer (0.25% sodium dodecyl sulfate, 0.05 N NaOH) and heated at 95°C for 15 min. The cell lysate was diluted by adding 180 μl of distilled water. The cell debris was spun down by centrifugation at 16,000 *g *for 5 min. Supernatants were used for PCR or frozen at -20°C until further use.

### Genotyping of isolates

The cultured vaginal and rectal GBS isolates were genotyped using RAPD-analysis with the RAPD Ready-to-Go beads (GE Healthcare. Buckinghamshire, UK) as described previously [29] with primer OPM1 (5' GTT GGT GGC T) at a final concentration of 2 μM, including 0.2 μM of fluorescent TET-labeled OPM1 primer. After 5 min at 94°C, 5 min at 35°C and 5 min at 72°C, reaction mixtures were cycled 30 times in a Veriti™ Thermal Cycler (Applied Biosystems, Foster City, Ca.), with the following conditions: 30 s at 94°C, 1 min at 35°C, and 1 min at 72°C, with a final extension period of 5 min at 72°C. Reaction vials were then cooled to 10°C and kept on ice until electrophoresis.

### Capillary electrophoresis

A volume of 11.9 μl of deionized formamide (ACE formamide, Lucron, De Pinte) was mixed with 0.6 μl of an internal size standard mixture containing 0.3 μl of the ROX-400 high-density size standard (Applied Biosystems, Foster City, Ca.) and 0.3 μl of Map marker 1000 size standard (BioVentures, Murfreesboro, Tn.). One microliter of OPM1-PCR product was added. The mixtures were denatured by heating at 95°C for 3 min and placed directly on ice for at least 10 min. Capillary electrophoresis was carried out using an ABI-Prism 310 genetic analyzer (Applied Biosystems) at 60°C, at a constant voltage of 1.5 kV, and at a more or less constant current of approximately 10 mA. Capillaries with a length of 47 cm and diameter of 50 μm were filled with performance-optimized polymer 4. Electropherograms were normalized using Genescan Analysis software, version 2.1 (Applied Biosystems).

### Data analysis

OPM1-PCR fingerprints were obtained as table files from the Gene Scan Analysis software and used in a software program developed at our laboratory [30]. Using these sample files composed of numbers, representing the length of the amplification fragments in base pairs, a distance matrix was calculated with the in-house software using the differential basepairs (dbp) and the Dice algorithm [30]. Clustering analysis was done with the Phylip software http://evolution.genetics.washington.edu/phylip.html, using the Neighbor Joining algorithm.

### Serotyping

A total of 122 GBS isolates from 36 pregnant women were serotyped, using the latex co-agglutination kit of Essum AB (Umea, Sweden), according to the manufacturer's instructions. This kit enables to differentiate between serotypes Ia, Ib, II, III, IV and V.

### Statistical methods

The McNemar test for correlated percentages was used to compare the sensitivity of the culture media.

## Results

For a total of 150 women, culture was carried out separately for both rectal and vaginal sites, using three culture methods, i.e. directly onto Columbia CNA agar, or indirectly, by subculturing onto Columbia CNA agar resp. Granada agar, following overnight incubation in LIM broth.

### Comparison of culture techniques

A total of 36 out of 150 pregnant women studied (24%) were colonized by GBS. Of 55 samples from which GBS was isolated, 22 were positive by direct inoculation on Columbia CNA agar, 55 by Lim broth enrichment with subculture on Granada agar and 45 by Lim broth with subculture on Columbia CNA agar, resulting in sensitivities of 40, 100 and 81% respectively (Table 1).

**Table 1:** Number of GBS-positive cultures detected by different culture media in separate vaginal and rectal specimens obtained from 150 pregnant women

Specimen	No. of positive GBS cultures detected by			
	Columbia CNA agar	Lim broth + Granada agar	Lim broth + Columbia CNA agar	Total no. of women colonized
Vaginal	11	28	24	28
Rectal	11	27	11	27
Total	22	55	45	36

Culture of vaginal specimens by direct plating on Columbia CNA agar was significantly less sensitive than culture in Lim broth with subculture on Granada agar or subculture on Columbia CNA agar (McNemar test, p < 0.0001). In addition, the culture of rectal specimens, direct plating onto Columbia CNA agar was significantly less sensitive than culture in Lim broth with subculture on Granada agar (p < 0.0001), which was more sensitive than culture on Lim broth with subculture on Columbia CNA agar (p = 0.0313).

### Carriage of GBS and genotyping

**Figure 1:**
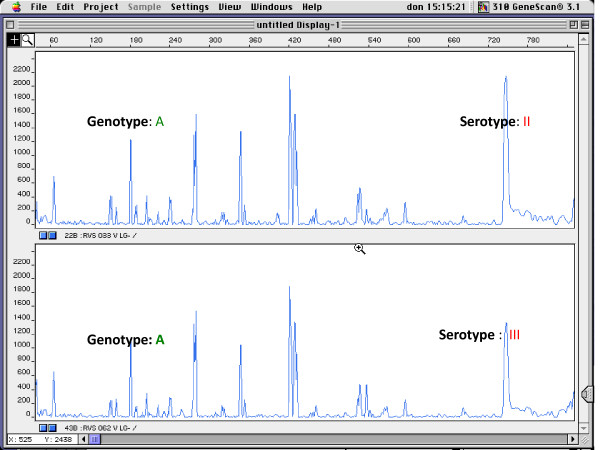
**Fingerprints of two isolates from two different subjects (RVS033 and RVS062) showing the same genotype and different serotypes**. x-axis: length of amplified DNA fragments expressed in bps. y-axis: peak height (intensity of DNA-fragment).

For the 36 women that were found positive, 9 (25%) carried GBS only in the vagina, 8 (22%) only in the rectum and 19 (53%) in both sampling sites (Table 1). Using three culture methods for two sampling sites, a maximum of 6 colonies per woman was picked, one from each of the positive culture plates. A total of 122 isolates were obtained (Table 2) of which 118 were genotyped using RAPD with primer OPM1 and analysis by capillary electrophoresis. The single isolates from four women were not fingerprinted, because there were no other isolates from the same subject to compare with. A tree was constructed after distance matrix calculation, and this revealed the presence of a total of 66 genotypes among the 118 isolates from 32 women for which fingerprinting was carried out. Only two subjects (RVS033 and RVS062) were found to carry strains of which the RAPD genotype was indistinguishable (Figure 1).

**Table 2:** Overview of genotyping and serotyping results for 36 women positive for GBS.

Number	Number	V DC	V LG	V LC	R DC	R LG	R LC
1	RVS143	-	**+**^a^/**V**^b^	-	-	-	-
2	RVS038	-	**A/III**	**A/V**	-	-	-
3	RVS071	-	**A/IV**	**A/IV**	-	-	-
4	RVS073	-	**A/III**	**A/V**	-	-	-
5	RVS109	-	**A/V**	**A/V**	-	-	-
6	RVS034	**A/III**	**B/Ib**	-	-	-	-
7	RVS035	**B/III**	**A/II**	**A/II**	-	-	-
8	RVS076	**A/Ib**	**B/Ib**	**B/Ib**	-	-	-
9	RVS148	-	**A/III**	**B/III**	-	-	-
10	RVS004	-	-	-	-	**+/Ia**	-
11	RVS051	-	-	-	-	**+/V**	-
12	RVS080	-	-	-	-	**+/IV**	-
13	RVS084	-	-	-	-	**A/NT**	**A/NT**
14	RVS021	-	-	-	**B/Ia**	**A/Ib**	**A/Ib**
15	RVS061	-	-	-	**B/V**	**A/NT**	**A/NT**
16	RVS120	-	-	-	-	**A/Ia**	**B/Ia**
17	RVS145	-	-	-	-	**A/Ia**	**B/V**
18	RVS017	-	**A/IV**	**A/IV**	-	**A/IV**	-
19	RVS047	-	**A/IV**	**A/IV**	**A/IV**	**A/IV**	**A/IV**
20	RVS106	**A/II**	**A/II**	-	**A/II**	**A/II**	-
21	RVS127	**A/IV**	**A/IV**	**A/IV**	-	**A/IV**	**A/IV**
22	RVS069	-	**A/NT**	-	-	**A/NT**	-
23	RVS039	-	**A/Ia**	**A/Ia**	-	**B/Ia**	**A/Ia**
24	RVS083	-	**A/III**	**B/Ib**	**A/III**	**A/III**	**A/III**
25	RVS114	-	**B/V**	**A/V**	-	**A/V**	**A/V**
26	RVS123	-	**A/NT**	**A/NT**	-	**A/NT**	**B/NT**
27	RVS027	**A/V**	**A/V**	**C/V**	**A/V**	**A/V**	**B/V**
28	RVS033	**A/III**	**C/III**	**A/III**	**A/III**	**B/III**	**A/III**
29	RVS064	**A/III**	**B/III**	**A/III**	**A/III**	**C/III**	**A/III**
30	RVS075	-	**B/II**	**A/II**	-	**C/II**	**A/II**
31	RVS110	**A/V**	**A/V**	**A/V**	**C/V**	**B/V**	**A/V**
32	RVS126	**A/NT**	**C/IV**	**A/NT**	-	**B/IV**	**A/NT**
33	RVS136	-	**A/NT**	**B/III**	-	**A/NT**	**C/NT**
34	RVS031	-	**D/III**	**A/III**	**B/NT**	**A/III**	**C/II**
35	RVS062	-	**D/II**	**A/II**	-	**B/II**	**C/II**
36	RVS074	**B/Ib**	**A/Ib**	**D/Ib**	**B/Ib**	**A/Ib**	**C/Ib**

Column headings: V DC: Vaginal sample, cultured directly on Columbia Agar, V LG: after Lim broth enrichment culture, plated onto Granada agar, V LC: after Lim broth enrichment culture, plated onto Columbia agar; R DC: Rectal sample, idem as for vaginal samples.

-: No GBS isolated.

^a^: Genotype: +: strain present, but not genotyped, A, B, C, D: genotypes numbered per individual.

^b^: Serotype: serotype number as determined by the Essum AB kit (Umea, Sweden). The following serotypes can be detected by this kit: Ia, Ib, II, III, IV and V. NT: nontypeable.

For 5 out of the 19 women from which both vaginal and rectal isolates were obtained, all the rectal and vaginal isolates were identical. For another 13 of these 19, at least one of the vaginal isolates had a similar genotype as one of the rectal isolates. Four women had two genotypes, seven had three genotypes and three had four genotypes. One of the five women with only rectal isolates had one genotype, whereas the other four had two genotypes. For the eight women with only vaginal isolates, four of them had only one genotype and the other four had two genotypes.

### Serotyping

**Figure 2:**
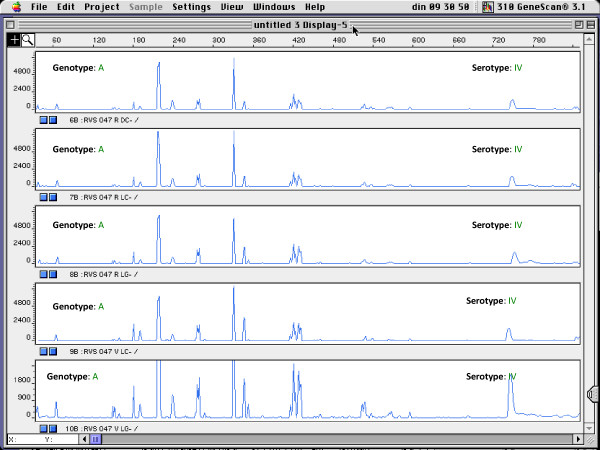
**Fingerprints of different isolates of subject RVS047 with the same genotype and the same serotype**. x-axis: length of amplified DNA fragments expressed in bps. y-axis: peak height (intensity of DNA-fragment).

**Figure 3:**
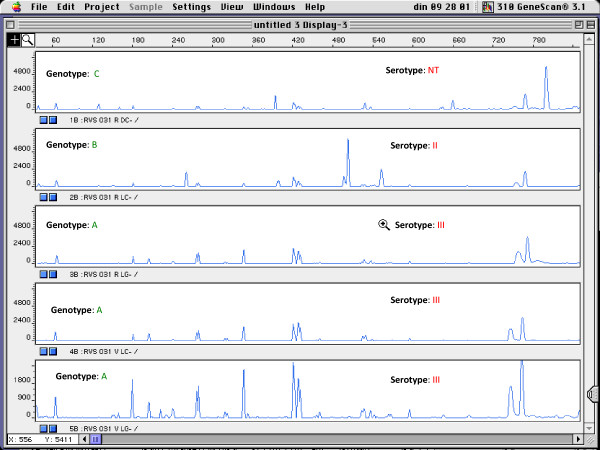
**Fingerprints of different isolates of subject RVS031 with different genotypes and different serotypes**. x-axis: length of amplified DNA fragments expressed in bps. y-axis: peak height (intensity of DNA-fragment).

In addition, all isolates were serotyped (Table 2). Seventeen isolates, from 7 subjects, were nontypeable. Furthermore, a rather equal distribution of the serotypes was found among the strains (Table 2). Eight women were found to carry isolates of the same genotype and serotype. An example is shown in Figure 2. In 11 subjects, only one serotype was present, although 2 to 4 genotypes could be found per subject. Nine subjects were found to carry isolates with different genotype and different serotype (Figure 3). The two isolates with the same genotype from the two different subjects belonged to different serotypes.

The frequency of different serotypes was as follows Ia: 10.4%, Ib: 10.4%, II: 10.4%, III: 20.8%, IV: 12.5, V: 20.8 and nontypeable: 14.5%. Overall serotype distribution among vaginal vs rectal isolates was comparable, with a rectal predominance of Ia, i.e. 1 vaginal isolate vs 5 rectal isolates and a vaginal predominance of III, i.e. 10 vaginal isolates vs 4 rectal isolates. The presence of *L. crispatus*, generally accepted to confer vaginal colonisation resistance to pathogenic organisms [31,32], did not seem to protect against vaginal *S. agalactiae *colonization, since a comparable number of women colonized only vaginally by *S. agalactiae *resp. only rectal by *S. agalactiae *were colonized vaginally by *L. crispatus*, i.e. 3/9 resp. 2/8. Six of the 19 women colonized by *S. agalactiae *both rectally and vaginally carried *L. crispatus *vaginally.

### Antibiotic susceptibility testing

GBS is considered homogenously susceptible to penicilline and amoxicillin. In case of allergy, second choice antibiotics are clindamycin or erythromycin. According to CLSI erythromycine can be tested with a simple disk test, although this is not done in our routine laboratory. Here we checked 40 isolates from 8 patients and found 26 isolates to be susceptible to both clindamycine and erythromycine, 3 to be clindamycine resistant and 11 to be resistant to both antibiotics. The susceptibility pattern of all strains was homogeneous for six subjects, despite genotypic differences among the isolates, whereas two subjects carried clindamycin resistant strains besides isolates susceptible to both antibiotics.

## Discussion

### Sensitivity and specificity of different culture techniques for the detection of GBS

In our study, only the combination of Lim broth and subculture on Granada agar enabled detection of all carriers, whereas direct inoculation onto Columbia CNA agar achieved a sensitivity of 40% and subculture of the Lim broth onto Columbia CNA agar detected 81% of the carriers.

Gil *et al*. [33] showed that in different studies the sensitivity of Granada agar ranged from 88.5 to 91.1%, that of Columbia CNA agar from 83.9 to 94.3% and that of Lim broth (to which 5% horse serum was added) was 63.5% when subcultured on Granada agar and 75% when subcultured on Columbia CNA agar. Bosch-Mestres *et al*. [34] showed that the use of direct inoculation onto Granada agar allowed fast detection for about 87% of carriers, where as the combination of Todd-Hewitt broth and subculture on Granada agar or Columbia CNA agar allowed detection of more than 99% of GBS carriers. Elsayed *et al*. [35] reported 79% sensitivity for direct inoculation onto Columbia CNA agar and incubation during 48 hours compared to 100% sensitivity for Lim broth with subculture on blood agar. It can be concluded from this study and others that the combination of broth enrichment (Todd-Hewitt or LIM broth) with subculture on solid medium, yields higher sensitivity than direct inoculation onto solid media.

Although no definite conclusions can be drawn with regard to the sensitivity of the different solid media, Granada agar has the advantage that it makes possible to provide overnight results by visual inspection of the presence of red orange colonies, that are produced exclusively by GBS. The red orange colonies are easily observed, even when few colonies are present or when GBS is mixed with other microorganisms, which are mainly other streptococci [33] . The colonies are so characteristic and unique that identification by antigen detection or the CAMP test is unnecessary. In this study, all red orange colonies were CAMP positive (data not shown). Claeys *et al*. [36] missed only two CAMP positive isolates on a total of 310 tested, which were not red orange pigmented on Granada. Blanckaert *et al*. [37] used a combination of Granada and Columbia blood agar for GBS screening and demonstrated that 6% of the samples positive for GBS on Columbia blood agar lacked red orange colonies on Granada agar. Notably, Granada agar does not detect non-pigmented isolates, and on blood-agar these non-hemolytic isolates are difficult to detect as well. As a result of that, non hemolytic, nonpigmented strains may have been missed. In conclusion, in our hands, the use of combination of Lim broth and subculture on Granada agar provided high sensitivity and specificity for detecting GBS in vaginal and rectal swabs from pregnant women.

### Epidemiology

The high prevalence of *S. agalactiae *colonization, i.e. 24%, as established in this study, is in accordance with results from other European studies that report colonization rates between 10% and 36% [38-43]. Thinkhamrop *et al. *[44] reported a prevalence of 7.1% (Myanmar) to 19.1% (Philadelphia), using Lim broth culture. Toresani *et al*. [9] found a prevalence of only 3.2% among 531 Argentinan pregnant women. Brimil *et al*. [38] showed equal carrier rates of 16% for GBS among pregnant and nonpregnant women and, based on the prevalence of GBS carriage, these authors concluded that strict adherence to the guidelines for prevention of GBS neonatal infection results in peripartal antibiotic prophylaxis in up to 20% of all deliveries.

Our data, are also in correspondence with other results on GBS prevalence in our country. Rectovaginal colonization with group B streptococci in Belgium is 13-25%. These data are based on different studies carried out by the Belgian reference laboratory for GBS in collaboration with the section of epidemiology of the Scientific Institute for Public Health (ISP-WIV, Brussels) [45]. For example, Blanckaert *et al. *[37] compared the results of GBS screening on Granada agar with those obtained using standard Columbia blood agar at two participating centers in Belgium. They reported GBS-positive culture results of 10-30% of pregnant women. The Flemish Study Centre for Perinatal Epidemiology evaluated GBS prevalence in Flanders and found an average colonization rate of 16% among Flemish pregnant women [46].

Manning *et al*. [41] found that the prevalence of GBS colonization was equally high among 241 women (34%) and 211 men (20%) living in a college dormitory. Sexually experienced subjects had twice the colonization rates of sexually inexperienced participants. Van der Mee-Marquet *et al. *[47] reported that the prevalence of carriage was 27% in women and 32% in men. The major positive body site was the genital tract (23% in women and 21% in men) and skin, throats, and anal margins were also positive in 2%, 4%, and 14%, respectively.

### Comparison between vaginal and rectal carriage

Brimil *et al*. [38] reported that for a total of 34 GBS positive pregnant women, 32% carried GBS only vaginal, 24% only rectal and 44% both rectal and vaginal, which compares well with our results, i.e. resp. 24%, 22% and 53%.

### Serotypic and genotypic diversity among GBS isolates

We studied the rectal and vaginal colonization with GBS of pregnant women attending the Ghent University hospital (Belgium) and the serotypic and genotypic diversity among the GBS isolates. Strong genotypic and serotypic heterogeneity was observed between women and for individual women, e.g., we found a total of 66 genotypes among 118 isolates (from 32 women), of which 4 women had up to 4 different genotypes in total (rectal + vaginal) and 14 women had up to 2 different genotypes vaginally.

Manning *et al*. [48] genotyped GBS isolates from vaginal-rectal swabs of women at two visits and documented a turnover in 8.3% of 126 women colonized both at 35-37 weeks of gestation and 6 weeks after delivery. Unfortunately, only one isolate was genotyped per visit. Taking into consideration the genotypic diversity per subject, as observed in our study, a turnover of 8.3% might be an overestimation. When the same subject, carrying e.g. genotypes a, b and c, is sampled at two different moments, and whereby on each occasion only one colony is picked, the detection of a different genotype may be interpreted as turn over, but it may be that a genotype a strain has picked at the first visit but a genotype b or c strain at the second visit.

Moreover, their conclusion that some clones are more likely to be lost should be interpreted with care, since their apparent disappearance might be explained by the presence of these clones in relatively lower numbers (compared to other clones), which reduces their chance of being picked at two separate occasions. To our knowledge, only one group studied genotypic diversity within individual women, by genotyping 15 randomly picked isolates for each of 30 women, albeit from mixed vaginal-rectal specimens [12]. In opposition to our results, these authors found a high degree of genotypic and serotypic homogeneity, i.e. for 29 of 30 women, all 15 isolates from each woman had the same serotype, and for 27 of 30 women, all 15 isolates had the same chromosomal *Sma*I-DNA restriction digest fingerprint. For the three women with different PFGE types, one had three different genotypes and two different serotypes. Another PFGE based study reported high stability of the GBS type for each woman, followed up to two years [40]. In accordance with our results, these authors found 30 different GBS isolates among 32 women, with only two women carrying isolates with the same genotype.

The high level of heterogeneity established in our study, compared to other studies, may be due to the different culture media that were used and to the use of separate swabs for sampling vagina and rectum for each individual woman. We found that strains of the same serotype recovered from different women were heterogeneous in DNA profiles. In accordance, *Sma*I-restriction digestion of chromosomal DNA and PFGE revealed high genotypic heterogeneity among both Zimbabwean serotype III and serotype V isolates [16], another study also reported different *Sma*I restriction types within serotypes II and V [12], and Fasola *et al*. [14] and Savoia *et al*. [49] showed that several genotypic lineages are present within the different serotypes.

Serotype switching is believed to occur within genotypic lineages [7,24,25], presumably by horizontal transfer of genes of the cps locus, i.e. of genes encoding the GBS capsular polysaccharide structure [50], and may be an explanation for our observation of the presence of two isolates in two different women with the same genotype, but with a different serotype.

Although MLST has become the standard method to study the population structure of GBS [22,24], more rapid and less expensive and laborious methods remain useful for carrying out single centre studies. We used RAPD-analysis in combination with high resolution capillary electrophoresis, which also makes possible immediate digitization of the fingerprints. Chatellier *et al*. [51] found that the simplest typing scheme of *S. agalactiae *was obtained by the combination of RAPD typing and serotyping (discriminatory index 0.97). Zhang *et al*. [20] found congruence between RAPD analysis and serotyping, on a limited number of strains. The findings of Toresani *et al*. [9], who found a total 16 RAPD profiles among 21 GBS isolates, from 17 women, and of Chatellier *et al*. [51], who identified 71 RAPD types among 54 unrelated *S. agalactiae *strains isolated from cerebrospinal fluid samples from neonates, point to the same genotypic diversity as observed in our study.

### General serotype distribution

The capsule of *S. agalactiae *has long been recognized as one of the most important virulence factors. Variations of the capsular polysaccharide structure allow the antigenic distinction of 13 different *S. agalactiae *serotypes, of which 9 are of clinical importance (Ia, Ib, II-VIII). Studies from the US and Europe show that the serotypes Ia, II, III, and V are found in 80-90% of all clinical isolates [4,48].

Serotype distribution among GBS isolates from pregnant women in our study was compared with that reported by others. Several studies indicate that serotype III is globally the most prevalent serotype, e.g. 29.2% of Israelian isolates [7], 28% of German isolates [38], 24.3% of Swedish isolates [39] and 33.2% of Czech isolates [42]. In accordance, we observed a frequency of 20.8% for serotype III isolates in our study, as the most frequent serotype.

### Antibiotic susceptibility

The finding that two out of 8 women carried isolates with different susceptibility to clindamycin indicates that when testing susceptibility for clindamycin several colonies should be tested, since colonies with different susceptibility may be simultaneously present.

## Conclusion

In summary, our study, including 150 pregnant women, confirmed the European prevalence of around 20% of GBS among pregnant women and the predominance of serotypes III and V among these women, but we established a higher genotypic diversity per individual than other studies, with up to four different genotypes among a maximum of 6 isolates per individual picked. Still, 18 of the 19 women with GBS from both rectum and vagina had at least one isolate from each sampling site with the same genotype. In our hands, the combination of Lim broth and subculture on Granada medium provided higher sensitivity than direct culture on Columbia CNA or Lim broth and subculture on Columbia CNA for GBS detection from vaginal and rectal swabs from pregnant women.

## Competing interests

The authors declare that they have no competing interests.

## Authors' contributions

NAE, RV, GC and MV participated in the development of the study design, the analysis of the study samples, the collection, analysis and interpretation of the data, and in the writing of the report. IT and MT participated in the development of the study design, the collection of the study samples, the collection, analysis and interpretation of the data, and in the writing of the report. BS and EDB participated in the analysis of the study samples and interpretation of the data. All authors read and approved the final manuscript.

## Pre-publication history

The pre-publication history for this paper can be accessed here:

http://www.biomedcentral.com/1471-2334/9/153/prepub
